# The Tired Pregnant Woman

**DOI:** 10.5811/cpcem.2021.5.51401

**Published:** 2021-09-27

**Authors:** Katie VanNatta, Nicole Yuzuk, David Trotter, Brandon Wisinski

**Affiliations:** * Midwestern University, Franciscan Health, Department of Emergency Medicine, Chicago, Illinois; † Saint Joseph’s University Medical Center, Department of Emergency Medicine, Paterson, New Jersey; ‡ Franciscan Health Olympia Fields, Department of Emergency Medicine, Olympia Fields, Illinois; § Midwestern University, Franciscan Physician Network Olympia Fields Clinic, Department of Emergency Medicine, Olympia Fields, Illinois

**Keywords:** CPC, pregnancy, hyperemesis gravidarum

## Abstract

**Introduction:**

Many pregnant women develop hyperemesis gravidarum. There are numerous gastrointestinal, genitourinary, neurologic, and metabolic causes to consider in this patient population.

**Case Presentation:**

This clinicopathological case presentation details the initial assessment and management of an 18-year-old pregnant patient who presented to the emergency department with a complaint of nausea, vomiting, fatigue, and intermittent bleeding.

**Discussion:**

This case takes the reader through the differential diagnosis and evaluation of the patient and the signs and symptoms, including her agitation and tachycardia, that led us to the correct diagnosis.

## CASE PRESENTATION (DR. KATIE VANNATTA)

An 18-year-old G1P0 female presented to the emergency department (ED) with a chief complaint of nausea, fatigue, and intermittent vaginal bleeding for the prior four days. She believed she was approximately two months pregnant but was unsure about her last menstrual period. She had not followed up with an obstetrician for her pregnancy, per chart review. The patient was given an informal diagnosis of hyperemesis gravidarum based on one previous ED visit when she had presented with emesis and was started on metoclopramide. She had been having six to eight episodes of emesis daily but denied emesis on the day of presentation. She reported light spotting but denied any tissue passage. She stated she had gone through one pad a day in the prior three days. Review of symptoms was positive for fatigue, nausea, vomiting, and vaginal bleeding. The patient had no chronic medical conditions or pertinent social or family history. She was taking metoclopramide 10 milligrams (mg) as needed for nausea and prenatal vitamins daily.

Upon arrival to the ED, the patient had a temperature of 97.9° Fahrenheit, heart rate of 160 beats per minute, a respiratory rate of 18 breaths per minute, oxygen saturation of 96% on room air, and blood pressure of 95/57 millimeters of mercury (mm Hg). On exam, she was tachycardic, had generalized abdominal tenderness, and appeared agitated with a flattened affect. She would answer questions appropriately but tersely with one word. A pelvic exam showed a closed cervical os with white vaginal discharge and a friable erythematous cervix that was tender to palpation, but no adnexal tenderness.

Initial laboratory results are listed in Tables 1 and 2. Other lab results included high sensitivity troponin of 14 (0–12 picograms per milliliter [pg/mL]), and quantitative human chorionic gonadotropin (hCG): >208,656 milli-international units per milliliter (m[IU]/mL). Her urinalysis showed large blood [negative], 3–5 red blood cell count (0–2 per high power field), 80 ketones (0 mg/deciliter), and moderate bacteria (none seen). An electrocardiogram was performed (Image) as well as a formal pregnancy ultrasound. The ultrasound demonstrated a single, live intrauterine gestation with crown-rump length corresponding to a gestational age of 10 weeks 4 days +/− 7 days.

With the abnormalities noted on initial labs, a right upper quadrant ultrasound was obtained showing only gallbladder sludge, without wall thickening, pericholecystic fluid, or common bile duct dilation, and a negative sonographic Murphy’s sign. Additionally, we obtained a serum lipase, acetaminophen, prothrombin time/international normalized ratio (PT/INR), activated partial thromboplastin time (aPTT), thyroid stimulating hormone (TSH), and alcohol level. The lipase, acetaminophen, and alcohol levels were all negative. The patient received two liters of normal saline with repeat vitals as follows: heart rate 132 beats per minute; blood pressure 127/77 mm Hg; and oxygen saturation 98% on room air. Following discussion with intensive care and obstetrics and gynecology, the patient was transferred to a tertiary care center for a higher level of care.

## CASE DISCUSSION (DR. NICOLE YUZUK)

When encountering nausea and vomiting in a pregnant patient, I think it best to break down the differential diagnosis into systems. There are numerous gastrointestinal, genitourinary, neurologic, and metabolic causes to consider in this patient population. One immediately thinks of gastrointestinal sources with vomiting, such as cholecystitis, pancreatitis, appendicitis, peptic ulcer disease, or small bowel obstruction. Additionally, there are pregnancy-related pathologies to think of as well, such as cholestasis or hemolysis, elevated liver enzymes, or low platelet count (HELLP) syndrome. It is reasonable to consider these diagnoses, especially cholecystitis and pancreatitis, given the patient’s liver abnormalities and abdominal tenderness. However, there is no mention of the right upper quadrant ultrasound being diagnostic, and the pain is not localized. Given her lab abnormalities, cholestasis of pregnancy and HELLP syndrome may initially be on the differential; however, because the patient is only in her first trimester it is too early for either of these diagnoses. From this list we can keep cholecystitis and biliary pathology because of lack of information regarding the patient’s ultrasound.

What about genitourinary causes of nausea and vomiting in pregnancy? Our list for this would include ectopic pregnancy, urinary tract infection or pyelonephritis, pelvic inflammatory disease, ovarian torsion, trophoblastic disease, and uremia. The patient’s workup ruled out many of these causes including ectopic/torsion, molar pregnancy, and kidney failure, but the emergency physician must still entertain the possibility of infectious causes. Her urinalysis was not convincing of urinary tract infection, and there was no mention of cervical motion tenderness; however, physical exam did reveal abnormal vaginal discharge and a friable cervix. Given these findings, it is reasonable to keep pelvic inflammatory disease on the differential, and one could argue that her vital signs are concerning for sepsis; however, she had no leukocytosis or other lab abnormalities that further supported infection.

Often, providers look to gastrointestinal causes as the most reasonable indication when someone is vomiting. Less commonly it could be the result of an intracranial process. Increased intracranial pressure could cause vomiting; possibilities to consider in the differential would be a central nervous system tumor or even intracranial hemorrhage. Although this is an important part of the differential, I can confidently eliminate this possibility for our patient because there was no mention of headaches, and aside from her agitation her neurologic exam was normal.

Metabolic causes of vomiting are always important to consider as they could range from a simple case of hyperemesis gravidarum to something more complex such as a thyroid disorder. Although I do think her prior hyperemesis diagnosis could be contributing to this patient’s pathology, in this case we must also consider thyroid disorders. Her presentation would make a hyperthyroid state more likely given her agitation and tachycardia. As we debate the possible explanations for her presenting complaints the only one that seems to tie everything together is a thyroid disorder. When thinking about hyperthyroidism in pregnancy, clinicians must determine whether the thyroid abnormality is from a primary disorder such as Graves’ disease, thyroiditis, or toxic adenoma or whether it is the result of gestational transient thyrotoxicosis. Graves’ disease is the most common cause of hyperthyroidism in pregnancy, but these patients often have a history of hyperthyroidism and exhibit other features consistent with the disease such as exophthalmos or goiter.^1^ This patient had no significant past medical history aside from the hyperemesis, and the physical exam did not reveal any abnormalities suggestive of thyrotoxicosis; therefore, although not impossible, it seems less likely to be related to these types of disorders.

This leaves us with gestational transient thyrotoxicosis. In pregnancy, thyroid physiology changes and the circulating hormone, human chorionic gonadotropin (hCG), can act like TSH on the thyroid gland, causing a fleeting increase in their thyroid function.^2^ Patients with hyperemesis gravidarum have a higher circulating amount of hCG and thus are at higher risk for this disorder.^3^ Patients with gestational transient thyrotoxicosis often do not show clinical features of hyperthyroidism (exophthalmos, goiter, tremor, etc), and the condition peaks at 10–12 weeks gestation. It is, therefore, reasonable when reviewing this case to think that the hyperemesis gravidarum incited this cause of hyperthyroidism and is the reason she is thyrotoxic despite the fact that these patients usually remain asymptomatic.^2^ The less common diagnosis hiding behind a presentation frequently seen in the ED is thyroid storm secondary to gestational transient thyrotoxicosis.

## CASE OUTCOME (DR. KATIE VANNATTA)

Before the patient was transferred from the ED to the tertiary care center, her TSH resulted as <0.01 (0.27 – 4.20 milli-international units per liter (mIU/L]) revealing that she was in thyroid storm. On the Burch-Wartofsky point scale (BWPS) the patient’s score for thyrotoxicosis was 45 points. Based upon the diagnosis, we obtained free thyroxine (T_4_) and triiodothyronine (T_3_) levels. After consulting with the tertiary care center, it was recommended to give 100 milligrams (mg) of intravenous (IV) hydrocortisone and withhold propylthiouracil due to her liver dysfunction. The patient’s additional labs resulted after she left the ED: PT: >134.0 (10.1–13.1s), international normalized ratio INR: >11.0 (INR: <1.1), APTT: >104.1 (25.0 – 36.0 s), T_3_, free (FT_3_): 5.55 (2.52 – 4.34 pg/mL), T_4_, free (FT_4_): 4.30 (0.55 – 1.60 ng/dL).

At the tertiary care center, the patient was admitted to the medical intensive care unit for four days to treat and control her thyroid storm in the setting of first trimester pregnancy. It was determined during her admission that her hyperemesis gravidarum was the underlying factor in her thyroid storm, liver failure, and vitamin K deficiency. Endocrine consult recommended starting hydrocortisone 50 mg IV three times a day, propranolol 10 mg every six hours, and methimazole once her transaminitis improved. She had a thyroid ultrasound and thyroid stimulating immunoglobulin testing, which were within normal limits. The patient’s daily T_3_/T_4_ was downtrending at the time of discharge, and she was sent home on 20 mg of prednisone for seven days. At the time of discharge, she had returned to her baseline.

## RESIDENT DISCUSSION (DR. KATIE VANNATTA)

Hyperemesis gravidarum is commonly encountered in the ED, but less known to emergency providers is the effect it has on thyroid function. These patients can have an increase in thyroid hormone level, but normally it is transient, unlike the patient in this case. The theory stems from the structural similarity of TSH and hCG, which triggers thyroid stimulation as hCG levels increase in pregnancy.^4^ Estrogens mediate this increase in sialylation that can reduce the clearance of thyroxine binding globulin resulting in increased levels of total T_4_ and T_3_.^4^ As pregnancy progresses, changes in albumin and free fatty acid concentrations can affect the binding of thyroid hormones to its carrier proteins resulting in lower serum levels of free T_3_ and free T_4_.^5^ Studies have shown that patients with a higher hCG concentration, such as those experiencing hyperemesis, may have an increased severity of vomiting and degree of thyroid stimulation.^6^ Most hyperemesis gravidarum patients who develop thyrotoxicosis have a transient course and do not require anti-thyroid medication.^3^

Thyroid storm is a rare, life-threatening condition in pregnancy which occurs in 1–2% of pregnant patients with hyperthyroidism. A hypermetabolic state caused by an excess of thyroid hormone, thyroid storm is diagnosed by a combination of signs and symptoms. It develops abruptly and affects the body’s thermo-regulatory, cardiovascular, nervous, and gastrointestinal systems, which in turn leads to multiorgan decompensation.^4^ The BWPS score (Table 3) can be used to help identify and predict the likelihood that biochemical thyrotoxicosis is thyroid storm.^7^ It is a quantitative diagnostic tool based on three major observations in patients with thyroid storm: continuum of end organ dysfunction; high variability of symptoms and signs between patients; and high mortality associated with missed diagnosis by assigning points for dysfunction of thermoregulatory, gastrointestinal, cardiovascular, and central nervous systems. Table 4 lists the recommendations for patients based on the BWPS score.^7^

Pregnant women with overt hyperthyroidism and thyrotoxicosis should be treated with a thioamide medication, such as methimazole or propylthiouracil, to minimize the risk of adverse outcomes.^8^ Historically, propylthiouracil was the preferred treatment for hyperthyroidism in pregnancy because it partially inhibits the conversion of T_4_ to T_3_ and crosses the placenta less readily than methimazole.^8^ The American Thyroid Association and the American Association of Clinical Endocrinologists recommend propylthiouracil therapy during the first trimester followed by a switch to methimazole at the start of the second trimester.^8^ This strategy helps avoid the rare complications of hepatotoxicity and methimazole embryopathy seen with use of methimazole early on in pregnancy.^8^ Corticosteroids are also a mainstay of treatment because of their effect on thyroid function.^9^ Hydrocortisone is commonly used since it inhibits the peripheral conversion of T_4_ to T_3_.^3^ Propranolol, a beta blocker, can also be used in pregnant patients with hyperthyroidism or thyroid storm. It is readily safe in pregnancy and can help with the symptoms as well as to decrease the conversion of T_4_ to T_3_.^9^

A link has been noted between hyperemesis gravidarum and thyrotoxicosis. Lin et al showed that women who were found to be in thyrotoxicosis with a TSH <0.02 also had severe liver dysfunction. Hyperemesis gravidarum can cause elevated liver enzymes because of the severe vomiting. Once the vomiting stops, the transaminitis usually returns to baseline.^10^ In a case report Shigemi et al described a link between maternal vitamin K deficiency with hyperemesis gravidarum. The case describes a woman with hyperemesis gravidarum who was found to have an increase in prothrombin time in the setting of mild liver dysfunction. This was complicated by malnutrition due to the severe vomiting most patients experience with hyperemesis gravidarum. 11

## FINAL DIAGNOSIS

Thyroid storm in the setting of first trimester pregnancy with acute liver failure and severe vitamin K deficiency due to hyperemesis gravidarum.

## KEY TEACHING POINTS

- Pregnant patients presenting to the ED with nausea/vomiting usually require only supportive management.
- When diagnosing a pregnant patient with hyperemesis gravidarum it is vital to assess the severity of malnutrition/dehydration to determine appropriate treatment and disposition.
- Less common complications linked to hyperemesis include hyperthyroidism, liver dysfunction, and vitamin K deficiency. Awareness of these conditions is important, as their identification can change disposition and management.
- Hyperemesis gravidarum usually causes a transient hyperthyroidism in early pregnancy that generally does not warrant treatment; however, in rare cases, thyroid storm can occur.
- Thyroid storm holds the highest mortality rate compared to the other severe complications of pregnancy.^5^
- Recommendations to treat thyroid storm in pregnancy include propylthiouracil in the first trimester and methimazole during the second and third trimesters.
- When presented with a similar, complex case, it is important that the emergency physician expand the workup and narrow a differential to make the less common diagnosis.

## Figures and Tables

**Image:**
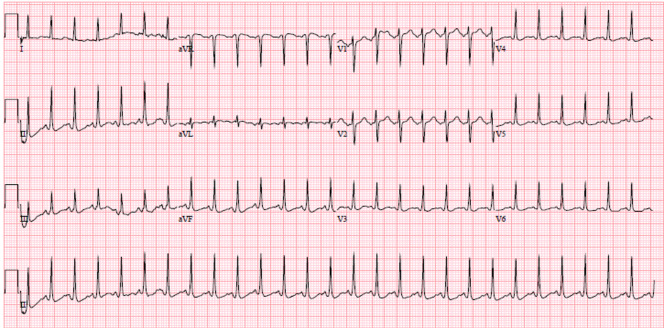
Electrocardiogram of an 18-year-old pregnant patient with emesis.

**Table 1:** Complete blood count with differential of an 18-year-old pregnant patient with emesis.

White Blood Cells	6.0 [ 4.0–11.0 10^3^/uL]
RBC	5.70 [3.63–5.04 10^6^/uL]
Hemoglobin	16.6 [12.0–15.3 g/dL]
Hematocrit	47.2 [34.7–45.1 %]
MCV	82.9 [80.0–100.0 fL]
MCH	29.1 [ 26.0–34.0 pg]
MCHC	35.1 [32.5–35.8 g/dL]
RDW	13.4 [ 11.9–15.9%]
Platelets	311 [150–450 10^3^/uL]
Neutrophil %	71.3
Lymphocyte %	19.6
Monocytes %	8.4
Eosinophil %	0.5
Basophil %	0.2
Neutrophil ABS CT	4.2 [1.7–7.7 10^3^/uL]
Lymphocyte ABS CT	1.2 [0.6–3.4 10^3^/uL]
Monocyte ABS CT	0.5 [0.3–1.0 10^3^/uL]
Eosinophil ABS CT	0.0 [0.0–0.5 10^3^/uL]
Basophil ABS CT	0.0 [0.0–0.2 10^3^/uL]
Mean Platelet Volume	10.4 [6.8–10.2 fL]

*RBC*, red blood cell count; *MCV*, mean corpuscular volume; *MCH*, mean corpuscular hemoglobin; *MCHC,* mean corpuscular hemoglobin concentration; *RDW*, red cell distribution width; *ABS*, absorbed; *CT,* cycle threshold; *uL*, microliter; *dL*, deciliter; *pg*, picogram; *g/dL*, gram per deciliter; *fL*, femtoliter.

**Table 2:** Complete metabolic panel of an 18-year-old pregnant woman with emesis.

Sodium	131 [133–144 mEq/L]
Potassium	3.0 [3.5–5.1 mEq/L]
Chloride	85 [98–107 mEq/L]
Carbon Dioxide	27.9 [21.0–31.0 mEq/L]
Anion Gap	18.1 [6.2 – 14.7 mEq/L}
Blood Urea Nitrogen	27 [7–25 mEq/L]
Creatinine	1.2 [0.6–1.2 mg/dL]
Calcium	9.8 [8.6–10.3 mg/dL]
Glucose	85 [70–99 mg/dL]
Alkaline Phosphatase Total	138 [34–104 U/L]
Total Protein	8.4 [6.4–8.9 g/dL]
Albumin	3.7 [3.7–8.9 g/dL]
Aspartate aminotransferase	302 [13–38 U/L]
Alanine aminotransferase	928 [7–52 U/L]
Total Bilirubin	2.4 [0.0–1.0 mg/dL]

*mEq/L,* milliequivalents per liter; *mg/dL*, milligrams per deciliter; *U/L,* units per liter; *g/dL*, grams per deciliter.

**Table 3:** The Burch-Wartofsky point scale.^7^

Temperature (° Fahrenheit)	<99	0
	99–99.9 (37.2–37.7)	+5
	100–100.9 (37.8–38.2)	+10
	101–101.9 (38.3–38.3)	+15
	102–102.9 (38.9–39.2)	+20
	103–103.9 (39.3–39.9)	+25
	>104 (>40.0)	+30
Central nervous system effects	Absent	0
	Mild (agitation)	+10
	Moderate (seizures, coma)	+20
	Severe (seizures, coma)	+30
Gastrointestinal-hepatic dysfunction	Absent	0
	Moderate (diarrhea, vomiting, abdominal pain	+10
	Severe (unexplained jaundice)	+20
Heart rate (beats per minute)	<90	0
	90–109	+5
	110–119	+10
	120–129	+15
	130–139	+20
	>140	+25
Congestive heart failure	Absent	0
	Mild (pedal edema)	+5
	Moderate (bibasilar rales)	+10
	Severe (pulmonary edema)	+15
Atrial fibrillation present	No (0) Yes (+10)	
Precipitating event	No (0) Yes (+10)	

**Table 4:** Recommendations based on the Burch-Wartofsky point scale.^7^

Score:	Suggestive of:	Recommendations:
>/=45	Highly suggestive of thyroid storm	Consider aggressive multimodal management in the intensive care unit
25–44	Impending thyroid storm	Consider starting thioamides, symptom management and consider intensive care unit monitoring
<25	Unlikely to be thyroid storm	Need to investigate diagnosis of thyrotoxicosis
